# Organotin Pollution in China

**DOI:** 10.1100/tsw.2002.136

**Published:** 2002-03-12

**Authors:** Qun-fang Zhou, Gui-bin Jiang, Ji-yan Liu

**Affiliations:** Research Center for Eco-Environmental Sciences, Chinese Academy of Sciences, P.O. Box 2871, Beijing, 100085, China

**Keywords:** organotin compounds, butylin compounds

## Abstract

A preliminary investigation of the occurrence of butyltin compounds was made in various environmental samples including water, sediment, sea products, and other commodities from China. Detection was carried out by the method of hydrogenation coupled with SPME or Grignard derivatization, followed by GC-FPD analysis. The results showed the universal existence of butyltin compounds in a wide range of tested samples, which was a potential danger for human health. Urgent control or management of organotin compounds is necessary to prevent the underlying hazard caused by this kind of pollutant.

## INTRODUCTION

With the wide use of organotin compounds during the last decades, growing public attention has been paid to their toxicity to nontarget biota and their potential danger to human health. Some organotin compounds, especially butyltin compounds, which were widely used in antifouling paints, have been put on the black and grey lists in several international treaties and were legislatively banned in antifouling paints from the late 1980s in most European and North American countries[1]. This was due to their all-pervading occurrence in various environmental samples as a result of flaking off of these paints from ships hulls, absorption by organic and inorganic particles, and subsequent deposition in sediments. A series of organotin-induced episodes, such as "stalinon affair"[2] have raised public cognition of organotins' toxicities.

In China, the approximate organotin consumption of 7,500 ton /year has caused widespread pollution. At the beginning of 1999, an unusual food poisoning affair caused by contaminated lard was found to be due to organotin pollution, with more than 1,000 victims affected. No legislative control or suitable management of organotin has been addressed yet. A comprehensive investigation on the occurrence and contamination of butyltin compounds in typical environmental samples in China is both urgent and necessary to accelerate the related legislative process.

A primary investigation on butyltin compound distribution in various environmental samples such as water, sediment, seafood, and other routine commodities are provided here to understand the current pollution of organotin compounds in China.

## MATERIAL AND REAGENTS

All butyltin standards including tetrabutyltin (TeBT, 96%), tri-n-butyltin chloride (TBT, 90%), di-n-butyltin dichloride (DBT, 97%), and n-butyltin trichloride (MBT, 97%) were obtained from Acros Organics (New Jersey).

Hydrogen derivatization reagent, sodium tetrahydridoborate ($NaBH_4$, 99.7%) was also provided by Acros Organics. The Grignard reagent n-propylmagnesium bromide (n-PrMgBr, 2.0M) was laboratory-prepared.

All other solvents and reagents used here were of at least analytical reagent grade.

### Instrumentation

A GC-9A gas chromatograph (Shimadzu, Japan) equipped with a fused silica capillary column coated with a film of 0.17 µm (HP-1; 25 m × 0.32 mm i.d.) and a laboratory-made flame photometric detector using quartz surface-induced luminescence (QSIL-FPD) was adopted for analysis of derived butyltin compounds.

The manual SPME device used was obtained from Supelco (Supelco, Bellefonte, PA). The optimized fiber was coated with 100 µm of polydimethysiloxane (PDMS) stationary phase.

### Sampling

Most of the sea water samples were collected near the east and south coastlines, where industry may bring heavy environmental pollution. Other water samples were from typical sea areas, lakes, or rivers, which might illustrate the whole organotin pollution status in Chinese aquatic system in some extent. Detailed distribution of butyltin was investigated in 50 sediment samples selectively collected near Shenzhen and Hong Kong, which could serve as a typical example of butyltin pollution in coastal sediment. The sea organisms included molluscs, crustaceans, and fish, and were collected in the local retail markets from six seashore cities located from southeast China to northeast China. The samples collected in Beijing retail market mostly came from such coastal cities as Tianjin, Qingdao, and Yantai. Some commodities including wine, liquid flavorings such as soy, yellow wine which were sealed in plastic packages, and a few special textiles were bought from shops.

### Analytical Procedure

A routine operation was performed for water, sediment, and other liquid samples by hydrogenation with $NaBH_4$, extraction with SPME, and analysis by GC-FPD, described in detail in our previous publications[3,4]. The reliability of this method was confirmed by approved spiked recoveries in water samples and content sediment recoveries in CRM 462 measurement.

Analysis of butyltin compounds in sea product samples was carried out by acidification with THF-HCl, extraction with 0.01% tropolone-hexane, Grignard propylation with (n-Pr)MgBr, drying and purification by a short column packed with anhydrous $NaSO_4$ and Florisil, followed by final separation and measurement by GC-FPD. The feasibility of the analysis procedure was confirmed by satisfactory recoveries in CRM477 determination[5].

## RESULTS

Widespread occurrences of butyltin compounds were found in all collected samples described above. Water, sediment, and sea product samples were mainly contaminated with TBT and its degradation products as the result of diffusion of TBT in antifouling paints, while other commodity samples were mainly contaminated with DBT and MBT due to their use as stabilizers and antiseptics.

### Water

The mean levels of tributyltin and its degradation products dibutyltin and monobutyltin in sea water samples were 93.8, 28.1, and 102.3 ng Sn/l, respectively, which were rather higher than the Environmental Quality Standards below 20 ng Sn/l issued in several west countries. Typically, serious butyltin contamination existed in the major ports of Dalian, Tianjin, and Qingdao. The results showed that fresh water in rivers and lakes were also polluted by butyltin compounds, especially in the areas of heavy shipping traffic or shipyards. The highest TBT level of 425 ng/l was detected in a ship port in Shanghai's Huangpu River. All these results were consistent with its use in some antifouling paints. As deleterious effects are found below few ng/l for some sensitive organisms[6], the current detected levels of organotin contamination in sea water and fresh water present significant hazards to aquatic organisms and even for human health.

### Sediment

The average levels in sediments from Shenzhen and Hong Kong were as follows: MBT, 104 ng/g; DBT, 36 ng/g; and TBT, 354 ng/g. It was reported that butyltin compounds would experience a long-term degradation in sediment because sediments were recognized as the ultimate sink for contaminants, such as organotins[7]. The accumulated species could be released into the water column through diffusive flux and resuspension/desorption phenomena[8], thus creating an ecotoxicological risk long after such anthropogenic sources have been banned. The high butyltin levels detected here would evidently affect the ambient aquatic ecosystem for a long period.

### Sea Products

As bioconcentration factors ranging from $10^2$ to $10^4$ were reported in various aquatic species including fish, crustaceans, and algae[9,10], the universal occurrence of butyltin compounds in aquatic system as described above would clearly cause butyltin accumulation in sea organisms. Thereby, it was of much interest to investigate butyltin levels in edible sea products. Small-scale surveys on the contamination status of butyltin compounds in commercial seafood collected from seven Chinese cities showed all-pervading existence of butyltins. The concentrations of TBT, DBT, and MBT ranged from <6.9 to 17,175; <5.2 to 692; and <7.8 to 40 ng Sn $g^{-1}$ wet weight, with a mean total butyltin level of 408 ng Sn $g^{-1}$. When compared with the data in corresponding water, the accumulation ratios of the pollutant in these organisms were as high as $10^3$ or more. Butyltin pollution levels varied with different sampling areas, wherein the most serious contamination existed in Dalian. A shellfish, Laternula limicola, accumulated extremely high levels of butyltin, especially TBT, which could suggest that this species was from a polluted area or had extremely high butyltin accumulation capability.

In addition, the usual manner of Chinese cooking or processing procedures was found to cause little effects on levels of butyltin compounds in seafood. All these results clearly indicated a potential danger for the health of people from seafood consumption.

Other butyltin contamination was also detected in some commodities, which could directly affect human health.

## DISCUSSION

It is known to all that, pollution, as the result of use of organotin compounds, particularly TBT-based antifoulants, has been of great concern due to persistence in sediment for periods of up to several years, the high bioaccumulation potential, and their high toxicity to marine organisms particularly molluscs[11,12] and marine mammals[13]. According to the results described above, their widespread use in conjunction with ships and fishing nets in China resulted in severe contamination of aquatic environments. Highly polluted sediment samples would be a second and long-term pollution source as the result of resuspension or desorption. High levels of contamination detected in sea products were the result of bioaccumulation and biomagnification, which would affect people's health directly. In addition, large amount of other organotin compounds, such as DBT and MBT, could be used as stabilizers or antiseptics for plastics, wood, and textiles, which could lead to contamination in routine commodities.

A number of studies have demonstrated the deleterious effects of TBT on nontarget organisms. Imposex, a condition in which male characteristics are imposed on genetic females, has been initiated by TBT exposure in several snail species[14]. In China, imposex was also found in the snail *Rapana venosa* as the result of the long-term effects of butyltin pollution. Although the effects of TBT on humans are not clear, shipyard workers exposed to TBT dust and vapors while repairing a submarine developed breathing problems, irritated skin, headaches, and stomachaches[15].

Serious problems of organotin pollution have already existed in China. Organotin compounds are still used in various fields because there is no legislative control or suitable management of these compounds in China. In view of toxicity effects, legislative action on the prohibition of organotin compounds is urgent and a more general survey on organotin pollution in China is necessary.

## CONCLUSION

A primary investigation of organotin compounds, especially butyltin compounds, in various environmental samples showed the widespread occurrence of this kind of pollution in China. An urgent review of legislation should be carried out in view of organotins' deleterious effects.

## ACKNOWLEDGEMENTS

This work was jointly supported by the National Natural Science Foundation of China and the Chinese Academy of Sciences.

## References

1. Chau, Y.K., Maguire, R.J., Brown, M., Yang, F., and Batchelor, S.P. (1997) *Water Qual. Res. J. Can*. **32**, 453.
2. Berman, E. (1980) *Toxic Metal and Their Analysis*. Heyden, London, p. 209.
3. Jiang, G.B., Liu, J.Y., and Yang, K.W. (2000) *Anal. Chim. Acta* **421(1),** 67–74.
4. Liu, J.Y., Jiang, G.B., Zhou, Q.F., and Yang, K.W. (2001) *J. Sep. Sci*. **24(1),** 459.
5. Zhou, Q.F., Jiang, G.B., and Liu, J.Y. (2001) *J. Agric. Food Chem*. **49,** 4287.
6. Fent, K. (1996) *Crit. Rev. Toxicol.* **26(1),** 1.
7. Unger, M.A., Macintyre, W.C., and Huggett, R.J. (1988) *Environ. Toxicol. Chem*. **7,** 907.
8. Dowson, P.H., Bubb, J.M., and Lester, J.N. (1993) *Appl. Organomet. Chem*. **7**, 623.

9. Hall, Jr., L.W. and Pinkney, A.E. (1985) *Crit. Rev. Toxicol*. **14,** 159.
10. Evans, D.W. and Laughlin, R.B. (1984) *Chemosphere* **13**, 213.
11. Gibbs, P.E., Bryan, G.W., Pascoe, P.L., and Burt, G.R. (1987) *J. Mar. Biol. Assoc. U. K.* **67,** 507.
12. Ritsema, R., Laane, R.W.P.M., and Donard, O.F.X. (1991) *Mar. Environ. Res*. **32**, 243.
13. Kannan, K., Senthilkumar, K., Loganathan, B.G., Takahashi, S., Odell, D.K., and Tanabe, S. (1997) *Environ. Sci. Technol*. **31**, 296.
14. Morcillo, Y. and Porte, C. (1998) *Trends Anal. Chem*. **17**, 109.
15. U.S. EPA (1985) Tributyltin Support Document.

## BIOSKETCH

**Qun-fang Zhou** is a student and doctoral candidate at the Research Center for Eco-Environmental Sciences, Chinese Academy of Sciences in Beijing. Her research is carried out on speciation analysis of organotin compounds in environmental samples using GC-FPD and GC-MS. With the combination of Grignard derivatization and organic solvent extraction, organotin compounds in environmental samples such as water, sediment and biota could be measured sensitively, quickly, and conveniently. Primary toxicity studies of tributyltin compound on a new fish *(Gobiocypris rarus*) were also carried out. The bioaccumulation of butyltin compounds in fish mussel was estimated and the ultrastructural changes were also observed through transmission electron microscopy. Ms. Zhou has been awarded First prize, Rector Scholarship, Chinese Academy of Sciences, 2000; and Second prize, Diao Scholarship, Chinese Academy of Sciences, 2000.
